# Comparative analysis of resistant and susceptible macrophage gene expression response to *Leishmania major* parasite

**DOI:** 10.1186/1471-2164-14-723

**Published:** 2013-10-22

**Authors:** Imen Rabhi, Sameh Rabhi, Rym Ben-Othman, Mohamed Radhouane Aniba, Bernadette Trentin, David Piquemal, Béatrice Regnault, Lamia Guizani-Tabbane

**Affiliations:** 1 Institut Pasteur de Tunis. Parasitologies medicales biotechnologies et Biomolecules, 13, Place Pasteur - B. P. 74., 1002 Tunis-Belvedere, Tunisia; 2 Institute for Advanced Computer Studies Center for Bioinformatics and Computational Biology (CBCB), University of Maryland, 20742 College Park, MD, USA; 3 Skuldtech. Cap Delta - ZAC Euromedecine II. 1682, rue de la Valsière, 34790 Grabels, France; 4 DNA Chip Platform, Genopole, Institut Pasteur de Paris, 25-28 rue du Dr Roux., 75015 Paris, France

**Keywords:** Microarray, Macrophages, *Leishmania*, Gene expression

## Abstract

**Background:**

*Leishmania* are obligated intracellular pathogens that replicate almost exclusively in macrophages. The outcome of infection depends largely on parasite pathogenicity and virulence but also on the activation status and genetic background of macrophages. Animal models are essential for a better understanding of pathogenesis of different microbes including *Leishmania*.

**Results:**

Here we compared the transcriptional signatures of resistant (C57BL/6) and susceptible (BALB/c) mouse bone marrow-derived macrophages in response to *Leishmania major* (*L. major*) promastigotes infection.

Microarray results were first analyzed for significant pathways using the Kyoto Encylopedia of Genes and Genomes (KEGG) database. The analysis revealed that a large set of the shared genes is involved in the immune response and that difference in the expression level of some chemokines and chemokine receptors could partially explain differences in resistance. We next focused on up-regulated genes unique to either BALB/c or C57BL/6 derived macrophages and identified, using KEGG database, signal transduction pathways among the most relevant pathways unique to both susceptible and resistant derived macrophages. Indeed, genes unique to C57BL/6 BMdMs were associated with target of rapamycin (mTOR) signaling pathway while a range of genes unique to BALB/c BMdMs, belong to p53 signaling pathway. We next investigated whether, in a given mice strain derived macrophages, the different up-regulated unique genes could be coordinately regulated. Using GeneMapp Cytoscape, we showed that the induced genes unique to BALB/c or C57BL/6 BMdMs are interconnected. Finally, we examined whether the induced pathways unique to BALB/c derived macrophages interfere with the ones unique to C57BL/6 derived macrophages. Protein-protein interaction analysis using String database highlights the existence of a cross-talk between p53 and mTOR signaling pathways respectively specific to susceptible and resistant BMdMs.

**Conclusions:**

Taken together our results suggest that strains specific pathogenesis may be due to a difference in the magnitude of the same pathways and/or to differentially expressed pathways in the two mouse strains derived macrophages. We identify signal transduction pathways among the most relevant pathways modulated by *L. major* infection, unique to BALB/c and C57BL/6 BMdM and postulate that the interplay between these potentially interconnected pathways could direct the macrophage response toward a given phenotype.

## Background

*Leishmania* lives as an obligate intracellular parasite within mammalian hosts. Host-*Leishmania* interactions are a complex interplay between a host’s defense mechanisms and the microorganism’s attempts to circumvent these defenses. The outcome of infection depends on parasite pathogenicity and virulence but also largely on the activation status and the genetic background of macrophages, the major target cells for parasite replication, also involved in the early events of pathogen infection.

Several studies have shown that *L. major* and other *Leishmania* species induces alteration in macrophages gene expression [1-6] and other have compared the effect of different *Leishmania* species including *L. major* on a given cell type [7]. However, only a recent study has highlighted the differences between the responses of murine macrophages from two inbred mouse strains to *L. amazonensis* infection [7].

Animal models are essential for a better understanding of pathogenesis of different microbes and the cutaneous leishmaniasis murine model has been widely used to characterize the response against *L. major.* In particular, these studies have capitalized on two different mouse strains with contrasted behavior in response to parasite infection: the BALB/c mice which develop severe lesions at the site of cutaneous inoculation [8] and the C57BL/6 mice with a self-healing lesion [9,10]. In this study we compared the transcriptomic signature of BALB/c and C57BL/6 derived macrophages and investigated whether susceptibility or resistance to *L. major* might reflect differences in macrophage responses to this parasite.

To distinguish the gene sets that belong to a known network of genes involved in biologically significant pathways, an *in silico* comparison was made using the KEGG database [11,12]. This analysis revealed shared and distinct expression profiles and showed that strains specific pathogenesis may be due to a difference in the magnitude of the same pathways but also to differentially expressed and potentially interconnected pathways in the two mouse strains derived macrophages.

## Methods

### Parasites

Tunisian strain of *L. major* promastigotes (MHOM/TN/95/GLC94 zymodeme MON25) were grown at 26°C in RPMI 1640, supplemented with 2 mM L-glutamine, 10% heat inactivated foetal calf serum, penicillin (100 U/ml) and streptomycin (10 mg/ml). Metacyclic rich fraction obtained using Ficoll gradient were used in all experiments. Briefly, stationary phase cultures of *Leishmania* were centrifuged at 5,000 g for 10 min at room temperature and resuspended in 2 ml of PBS. The cell suspensions were then loaded onto a Ficoll gradient composed, from the bottom of 2 ml of 20%, 5 ml of 10% and 5 ml of 5% Ficoll diluted in PBS. The gradient was next centrifuged at 1,300 g for 10 min at room temperature. The metacyclic promastigotes were recovered on the top of 10% Ficoll layer.

### Cells isolation and culture

BALB/c and C57BL/6 mice (Elevage Janvier) were killed and hind legs removed for BMdM isolation. Briefly, femurs and tibias were flushed with RPMI 1640 using a 25-gauge needle. Contaminating erythrocytes were lysed through the addition of Geys lysis solution (ammonium chloride 1.5 M, EDTA 0.1 mM, pH 7.3). All cells were incubated in T75 culture flasks at 1.5 106 cell per ml in RPMI 1640 media supplemented with 2 mM L-glutamine, 10% heat inactivated foetal calf serum (Perbio science, Brebières, France), penicillin (100 U/ml) and streptomycin (10 mg/ml) and 80 ng/ml M-CSF (Peprotech, Neuilly sur Seine, France) overnight for stromal cell elimination. Non-adherent, immature macrophages were transferred to fresh culture-treated Petri dishes (Nunc, USA) and grown for 7 days, with re-feeding on day 3, to induce macrophage differentiation. BMdM purity was analyzed through the evaluation of phenotypic expression of specific macrophage subset surface marker (F4/80) by Flow cytometry. Generated macrophages were assessed by flow cytometry for expression of F4/80 (80-90% were positive).

### Ethics statement

All mouse work was done according to the directive 86/609/EEC of the European parliament and of the council on the protection of animals used for scientific purposes. Approval for *mice* experiments was obtained from the ethic committee of Institute Pasteur of Tunis with ethics approval number 1204.

### Cells infection

10^6^ BMdM Cells were seeded in 1 ml complete media on 24 well plates and subjected to adhere overnight at 37°C in 5% CO2. They are afterwards, incubated at a parasite to cell ratio of approximately 10:1 with Ficoll purified metacyclic promastigotes of *L. major*. After the desired time of incubation, the extracellular parasites were washed out and the cells were harvested to prepare samples. The macrophages were fixed, Giemsa-stained and counted to calculate the number of amastigotes per 100 macrophages to insure for homogenate cell infection under the different conditions.

### RNA isolation, microarray hybridization and normalization

The RNA isolation and quantification, the hybridization to the GeneChip Mouse Gene 1.0 ST array (Affymetrix, Santa Clara, CA) were performed as previously described [6]. Each infection and control time points were performed in triplicate, using different preparations of BMdMs, and processed independently to give three biological replicates. QC analysis was performed before and after normalization using BoxPlot of total intensities, MAPlots for all replicates and PCAplots. All microarrays of this study passed the quality control.

The intrachip and interchip normalisation were performed as previously described [6]. Expression analysis used the R Bioconductor package Limma [13] to identify genes that met statistical (*P* < 0.05 after adjustment according to the method of Benjamini and Hochberg and fold-change criteria (at least a 1.5-fold change) for differential expression using the following contrasts: macrophages infected with live parasites at a given time point versus non infected macrophages incubated with vehicule (media) for the same time. The same contrast was used for heat-killed *L. major*-infected macrophages. Macrophage genes modulated during the kinetics were detected.

In accordance with MIAME (Minimum Information About a Microarray Experiments) regulations [14], all data were deposited into GEO (Gene Expression Omnibus) database at http://www.ncbi.nlm.nih.gov/geo under the accession number GSE31995 and GSE31996.

#### Quantitative real time PCR

Transcripts significantly modulated by *Leishmania* infection over the time were identified and a subset of these genes confirmed by reverse-transcription quantitative real-time PCR (RT-qPCR) as previously described [6]. The same RNA samples were used for both affymetrix microarray analysis and qRT-PCR experiments.

### Data pre- processing

Original data represented as matrix was subject to a normalization pipeline, which consists in i) merging different triplicates for each condition into a single data point reducing the matrix columns from 45 columns to 15 columns and one additional column for non infected at t = 0 h. ii) To avoid gene duplicates, we merged probes with same gene ID which automatically shrinks the matrix so that all rows of probes from the same gene are merged into one average row using only gene ID as unique identifier. iii) In order to remove systematic variations that may occur because of reasons other than biological differences between RNA samples, a Quantile Normalization has been done on the expression data. iv) A fold change of 2 was used as a cutoff to further reduce the data and keep highly differential genes and a final standardization of the data was done on the mean (0) and standard deviation (1) of the final gene set on the expression matrix.

### Conditions comparison

To compare conditions in order to extract highly differentially expressed genes, our strategy consists on the use of a supervised grouping using *T*-test as a statistical approach to extract significantly variable genes. Similar strategy was used for either BALB/c or C57BL/6. We first compared the group of uninfected conditions (NI) (at the different time points (1, 3, 6, 12 and 24 h)) to the infected group (P) (at the same time points). Similar comparison was performed between the parasite infected (P) and killed parasite (Kp) infected group. Selected probes obey the p-value cutoff of 0.05 using FDR as multiple tests correction. The outcome of these steps is groups of genes that are down-regulated or up-regulated depending on their expression values.

### Pathway analysis

In order to locate in which pathway our up-regulated and down-regulated genes are enriched, we relied on the usage of KEGG pathway for this analysis. We used a stringent p-value cutoff of 1E-4 and the condition of having at least 4 genes within each enriched pathway. The results consist in a set of enriched pathways for up-regulated genes and down-regulated genes. Gene lists of each pathway found are extracted for further analysis. We run an additional pathway enrichment analysis based on gene occurrence in both BALB/c and C57BL/6 as well as genes that are unique to each of them. The goal behind this additional analysis is to detect pathways that are specific to each mice strain under the same infection conditions. The analysis of specific genes may inform on interconnected pathways that may be activated or deactivated depending on the gene sets involved.

### Protein-protein interaction networks

In order to discover relationships between genes products in our up-regulated and down-regulated sets we used STRING DB as it contains an accurate and updated data on physical and functional interactions. STRING also allows to predict activation/repression relationships between different nodes of the same graph [15].

In furtherance of highlighting relationship between differentially expressed genes and corresponding pathways, we used all up-regulated genes in BALB/c and C57BL/6 and studied genes interaction within the same pathway and genes interactions across different pathways. The analysis was done using GENMAPP-CS cytoscape plugin (http://www.genmapp.org/beta/genmappcs/). Orphan nodes were removed from the network to highlight the direct interactions between different genes.

## Results

### Infection rates and parasites load were equivalent

We first established cell culture and infection conditions to ensure that the levels of infection of the bone marrow derived macrophages (BMdM) isolated from the two mice strains were equivalent. The same cell/parasite ratio (1:10) was found to give similar infection rate and parasite load. Light microscopy on Giemsa stained chamber slides of infected BMMs showed that the percentage of infected cells and the mean amastigote loads were comparable between *L. major* infected C57BL/6 and BALB/c BMdMs in each of the three experimental replicates used in this study (Table 1). Subsequent microarray analysis was carried out on each of the three biological replicates.

**Table 1:** **Equivalent parasite loads following infection with *L. major *** **promastigotes**

		**% of infected cells at 24 h point time**	**Number of** ***L*.** ***major*** **parasites per cell**				
**Repeat experiment number**			**0**	**1**	**2**	**3**	**>4**
1	Balb/C	91	9	11	14	19	47
	C57BI/6	91	9	15	15	16	45
2	Balb/C	87	13	17	16	14	40
	C57BI/6	91	9	6	16	17	52
3	Balb/C	85	15	17	20	13	35
	C57BI/6	86	14	13	21	18	34

BMdMs were plated on chamber slides and infected for 24 h; washed thoroughly; The percentage of cells infected and the mean number of parasites per cell were determined following staining with Giemsa according the manufacturer’s instructions.

### Microarray analysis

**Figure 1:**
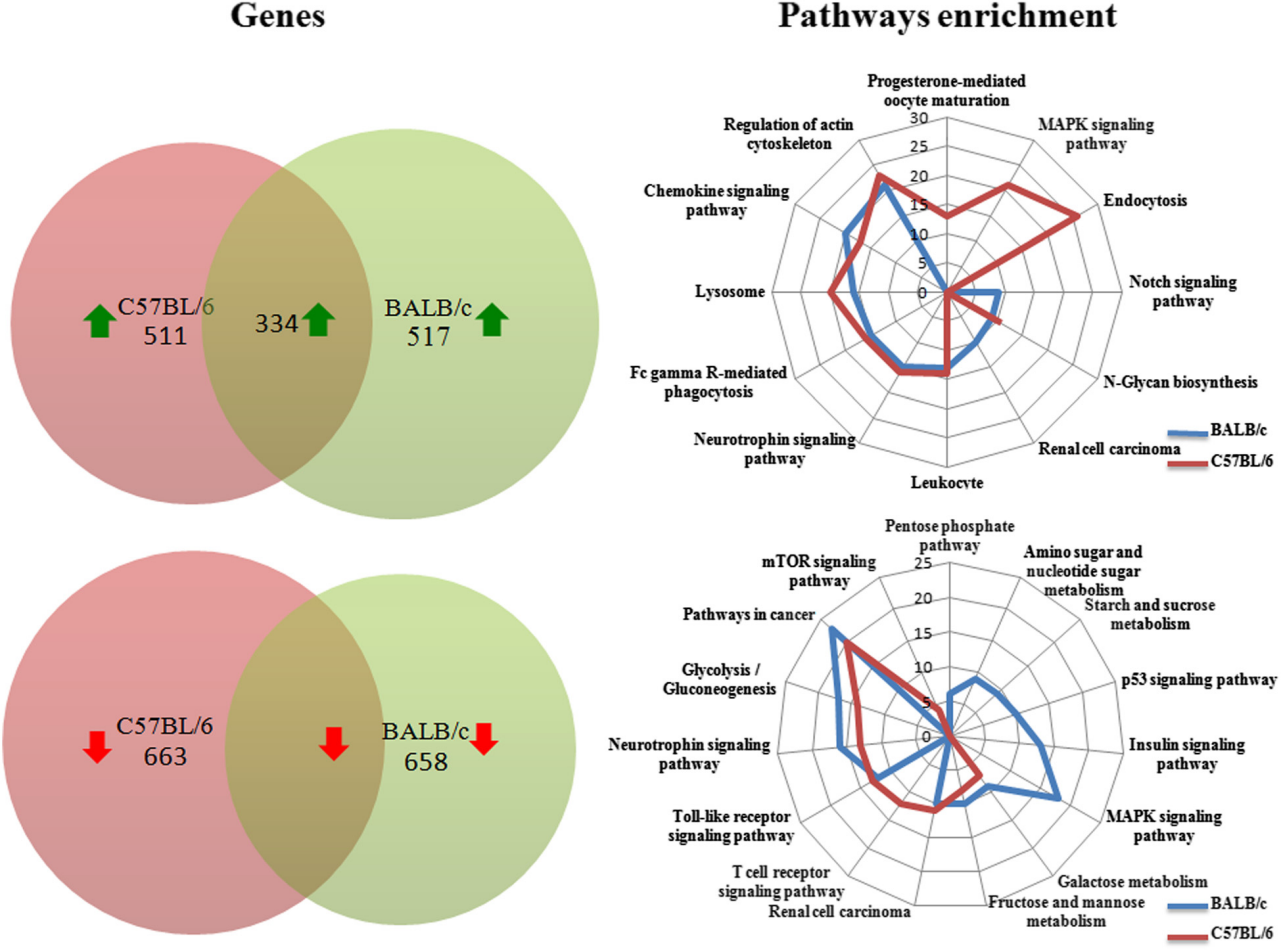
**Comparison of significant host genes and pathways differentially expressed in *Leishmania-*infected bone marrow derived macrophages.** Venn diagrams comparing up- or down-regulated genes (≥ 2-fold, *p* < 0.01) and radar plot reporting canonical pathways predicted as significantly modulated (*p* < 0.05) in *Leishmania* infected BALB/c and C57BL/6 derived macrophages. Metabolic pathways have been omitted in this representation.

GeneChip Mouse Gene 1.0 ST arrays were used to analyse global changes in gene transcripts to generate a pool of genes that was statistically significant (p-value < 0.05) and a fold change cut-off of 2. Of the 18 899 genes represented on the array, our analysis of mRNA expression in mice BMdM infected by live parasites showed that a total of 1175 genes were expressed differentially over the time course (with 658 down- and 517 up-regulated) in BALB/c BMdM and approximately the same number of genes (1174) was differentially modulated (663 down- and 511 up-regulated) in C57BL/6 BMdM. The microarrays analysis also revealed that as a result of *Leishmania* infection, the expression of 768 genes were shared between BALB/c and C57BL/6 BMdMs, with 434 genes down-regulated and 334 up-regulated (Figure 1). The relative expression of selected differentially expressed genes from the microarray data was further examined by RT-qPCR on the same samples that those analyzed by microarray analysis. Data from RT-qPCR analysis (Additional file 1: Table S1) were consistent with the results obtained by microarrays, albeit with magnitudes different from, and often higher than, those recorded by the microarray analysis.

Further analysis showed that 768 genes among the commonly altered in BALB/c and C57BL/6 BMdMs in response to infection, fall into 45 KEGG pathways. The genes unique to BALB/c correspond to 22 and those unique to C57BL/6 correspond to 25 pathways with a p-value less than 0.05 (Additional file 2: Table S2).

### Shared expression profiles in susceptible and resistant *Leishmania* infected BMdM and strains specific pathogenesis

Functional analysis determined that the shared genes were primarily associated with metabolic pathways (including glycolysis/Gluconeogenesis, Galactose, Fructose and mannose metabolism, and N-Glycan biosynthesis). Analysis and biological validation of a set of those metabolic pathways have been previously reported [6]. *L. major* also modulates different other genes implicated in Fc gamma R-mediated phagocytosis, chemokines, and Toll like receptor signaling pathway. The expression of different genes involved in host cell defense pathways and especially in iron metabolism is also altered by the infection. Indeed, almost all the intracellular pathogens require iron to develop a productive infection and restricting the availability of iron is considered as an important strategy for defense against infections. *L. major* inhibits the mRNA expression of the genes encoding transferring, holotransferrin receptor and Ferroportin 1 (Slc40a1) which suggest that the parasite limits the uptake and the export of iron. *L. major* also enhances the induction of the mRNA of Nramp2 a protein implicated in the iron efflux from the endosomes suggesting more iron accumulation inside the cytosol. Otherwise, in both susceptible and resistant *Leishmania major* infected macrophages, the parasite induces the transcription down-regulation of most lysosomal proteins. These include different Glycosidases (Gusb, Galc), Sulfatases (Sgsh), membrane proteins (Laptm5, Lamp1), most V-ATPases and others lysosomal proteases such cathepsin.

We further analyzed the expression and kinetics of a panel of cytokines and chemokines during *L. major* infection in a comparative study of genetically resistant C57BL/6 and susceptible BALB/c mice. Among the chemokines responsible of leukocytes recruitment, *L. major* induces the mRNA expression of Ccl2 (MCP1), macrophage inflammatory protein-1α (Mip-1α/Ccl3) and Mip-1β/Ccl4. The infection also activates the transcription of Cxcl1, Cxcl2 that regulate the influx of PMNs, Cxcl3 that controls migration of monocytes, Cxcl9 the Th1-attracting protein and the interferon-γ - inducible protein-10 (IP-10/Ccxl10). The expression of Cxcl1, Cxcl2 and Cxcl3 mRNA induced by both live and killed parasites is not modulated to the same extent in the two mice strains BMdM. Indeed, as assessed by qRT-PCR, the mRNA induction of these chemokines is respectively six, five and four times higher in the C57BL/6 BMdM (Additional file 1: Table S1). Moreover, in the macrophage derived from susceptible mice, the transcription induced by live parasites seems actively repressed when compared to the one induced by killed parasites. The mRNA expression of Ccl3 is similarly regulated. *L. major* promastigotes induce rapid and transient expression of murine Ccl2 that besides attracting monocytes and macrophages, can attract other cells such as NK and DCs expressing the chemokine receptor Ccr2. However, the infection represses the transcription of Ccr2 receptor. By contrast, parasites activate the mRNA expression of Ccrl2 in macrophages derived from the two mice strain. This expression is however 5 times more important in resistant mice. The mRNA expression of Cxcl9, slightly increases in response to *L. major* during early infection in BMdM derived from the two mice strains. However, while this expression is quiet back to the baseline 24 hpi in BALB/c BMdM, it starts to significantly be enhanced beginning from 12 hpi in the C57BL/6 BMdM.

### Signal transduction pathways are among the most relevant pathways unique to BALB/c and C57BL/6 macrophages

We next identified differentially expressed genes unique to BALB/c and C57BL/6 derived macrophages and performed enrichment analysis of these genes based on KEGG database (Additional file 2: Table S2). Pathways with *p-*values less than 0.05 were considered statistically significant. This analysis clearly identifies signaling pathways among the most relevant pathways modulated by *L. major* promastigotes. Indeed, the results show that down-regulated genes unique to macrophages derived from resistant mice were related among others to MAPK signaling pathways whereas a set of up-regulated genes were involved in the mTOR signaling pathway, Erbb and Insulin signaling pathways (Additional file 2: Table S2). Among the down-regulated genes unique to susceptible mice we listed those implicated in the Notch and chemokine signaling pathways. The up-regulated genes unique to BALB/c derived macrophages are implicated in p53 signaling pathway (Additional file 2: Table S2). Among these p53-dependent genes modulated by *L. major*, we found the mouse double minute 2 (Mdm2) an inhibitor of p53, the insulin growth factor 1 (Igf1), some genes implicated in cell cycle genes such Gadd45a or b, and genes implicated in the apoptotic pathway (Bid).

**Figure 2:**
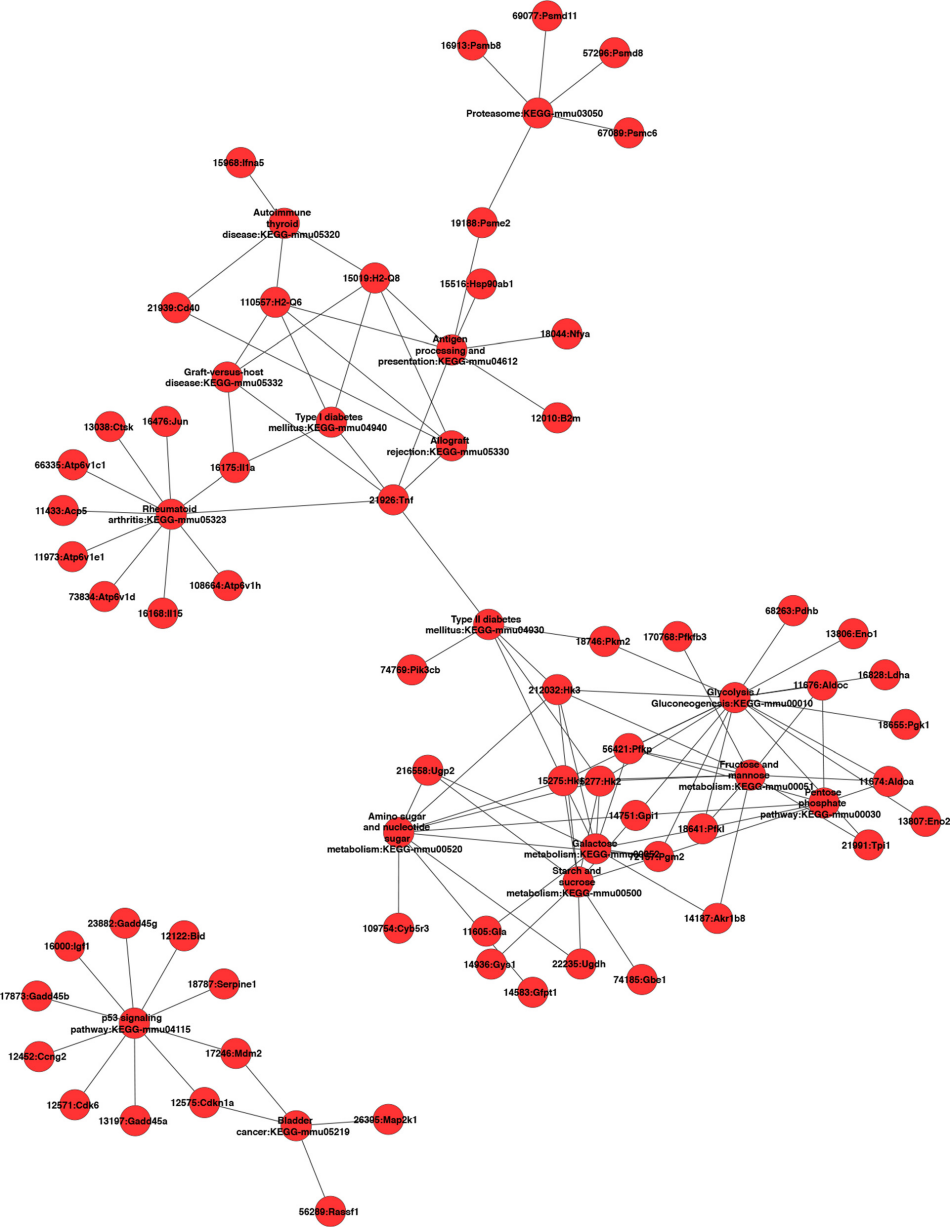
**Cytoscape plugin was used to analyze within Balb/c up-regulated genes, genes interaction within the same pathway and across different pathways.** Orphan nodes were removed from the network to highlight the direct interactions between different genes.

**Figure 3:**
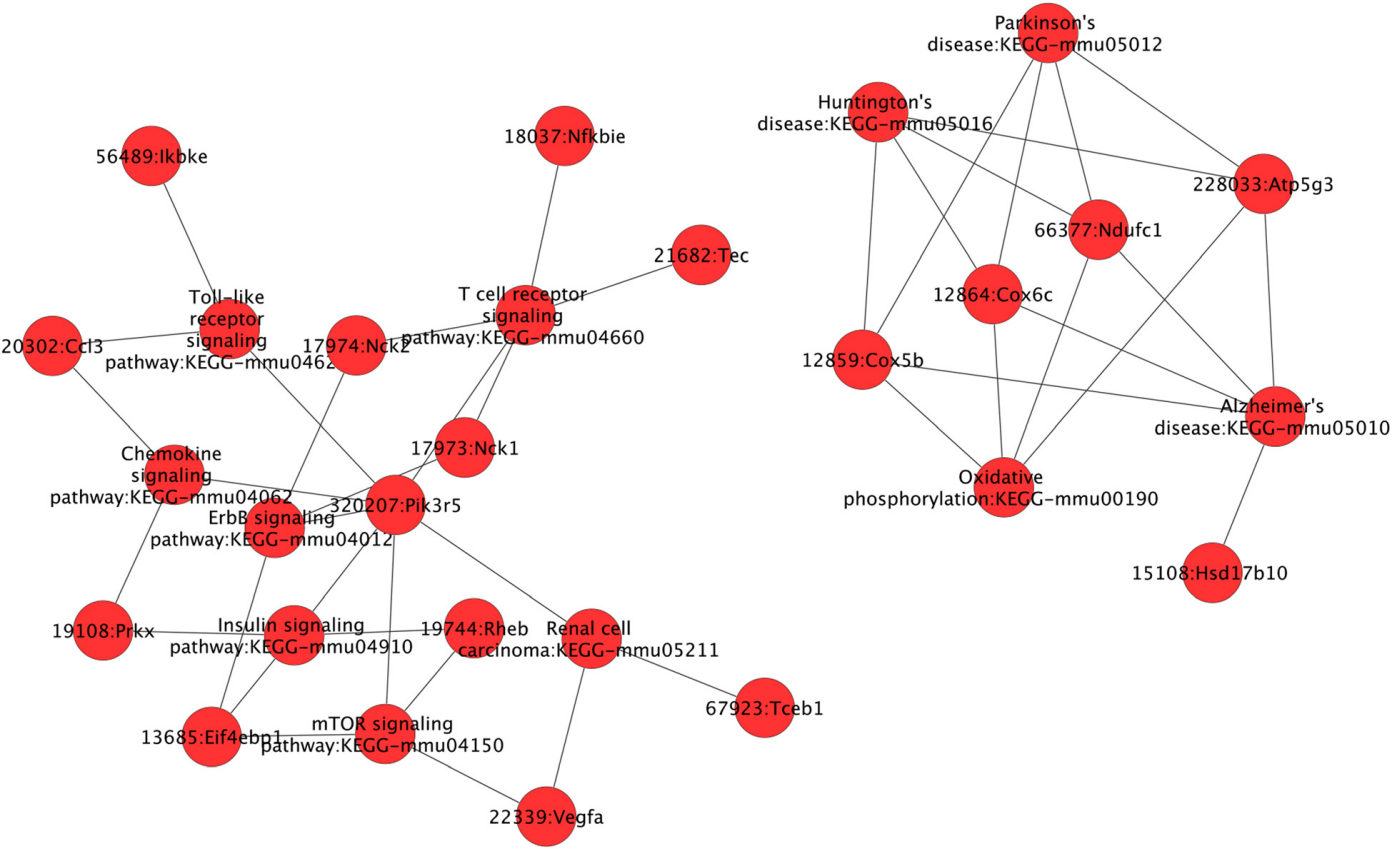
**Cytoscape plugin was used to analyse within C57Bl/6 up-regulated genes, genes interaction within the same pathway and across different pathways.** Orphan nodes were removed from the network to highlight the direct interactions between different genes.

Furthermore, we investigated whether, in a given mice strain derived macrophages, these coordinately regulated unique genes may be interconnected. We focused on up-regulated genes and up-loaded on GENMAPP-CS the lists of the genes unique to BALB/c and C57BL/6 derived macrophages. This analysis shows (Figure 2) that after the removing of orphan nodes, the genes (and pathways) identified to be significantly induced by *L. major* in macrophages derived from susceptible mice, mostly unique to BALB/c appeared to be interconnected except for the p53 signaling pathway (Figure 2). These fall into several pathways including glycolysis/Gluconeogenesis (AldoC, Eno2, Pdhb), antigen processing, cellular pH regulation (VATP-ases, Cathepsin), immune response (TNFα, IL-1α). Our results also show that several genes and pathways induced by *L. major* in C57BL/6 macrophages are linked to the mammalian Target of Rapamycine (mTOR) signaling pathway (Figure 3). These pathways include the chemokine, the toll like receptors and the insulin signaling pathways.

**Figure 4:**
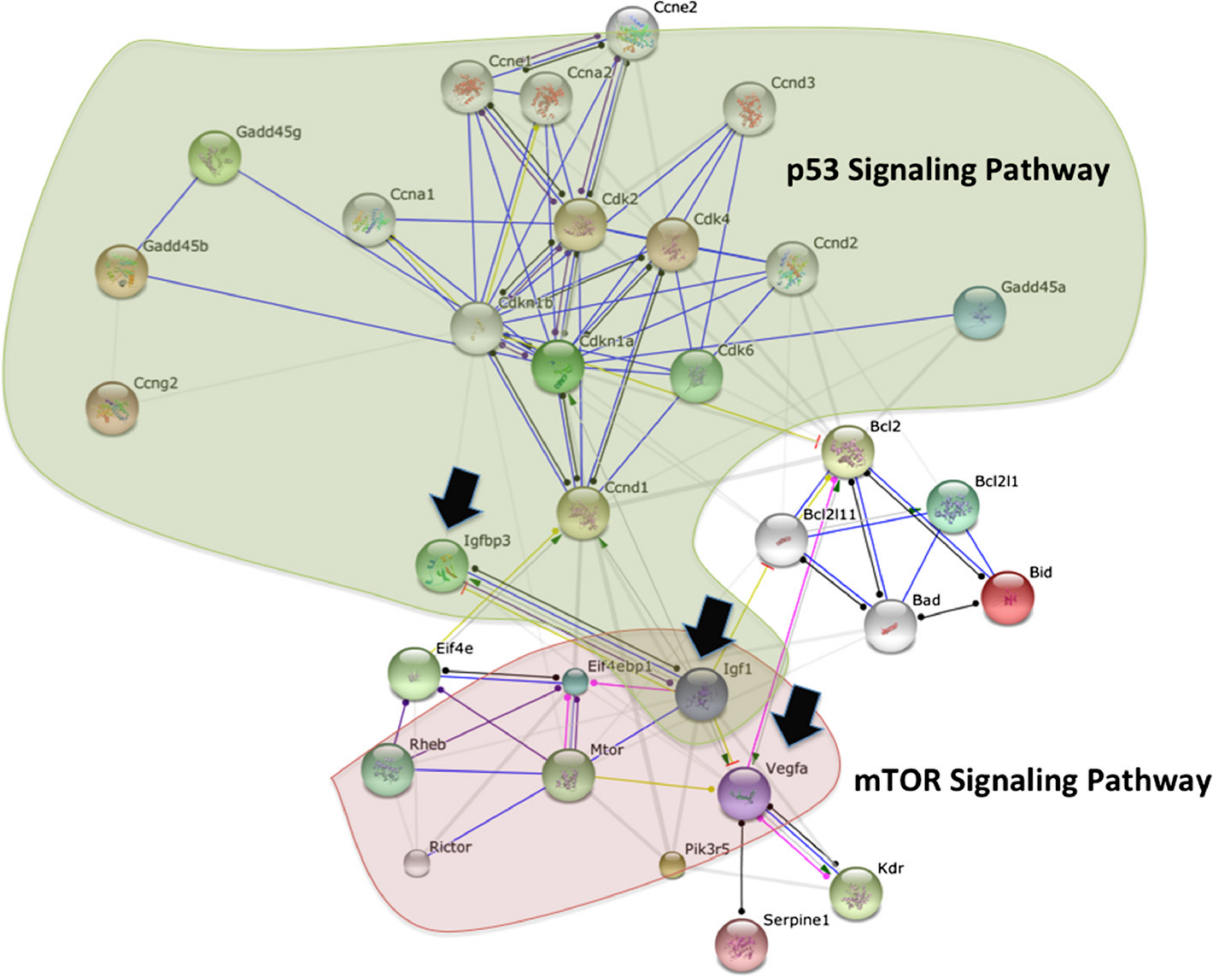
Interaction network built using STRING database.

As p53 and mTOR signaling pathways are known to be interconnected, the list of *L. major*-activated genes belonging to these two pathways were uploaded on STRING database. As illustrated in Figure 4, protein-protein interaction analysis, show that these two pathways are independent with Igf1, a p53 target gene, showing up as a major node in this network.

## Discussion

We compared transcriptomic signature of susceptible and resistant bone marrow derived macrophages in response to *L. major* infection. The analysis identifies both shared and unique set of transcribed genes.

Evidence for early inflammatory response have been presented for either human [16] or murine [6] macrophages exposed to *L. major*. Host defense is highly dependent on mobile leucocytes and cell trafficking is largely mediated by the interactions of chemokines with their specific receptors expressed on the surface of leucocytes. Chemokines and their receptors play a critical role in the regulation of leukocyte recruitment during leishmaniasis. Chemokine receptors work in concert or succession to successfully recruit to sites of inflammation, effectors cells such PMNs, macrophages and NK cells in order to combat skin infection with *Leishmania*[17]. Chemokines also have roles in adaptive immunity, in macrophage activation and parasite killing.

Taken together, our results show that the expression of these chemokines and some of their receptors is induced in both mice strain derived macrophages with however, a more rapid and heightened expression in C57BL/6 BMdMs. Differential expression of chemokines and chemokine receptors may lead to difference in immune cells influx. This difference in inflammatory cells recruitment can affect T cells activation which could explain differences in resistance. In accordance with this hypothesis and using electron microscopy combined with enzyme-histochemical methods, qualitative and quantitative differences in the pattern of infiltration at the site of infection have been reported between *L. major* infected BALB/c and C57BL/6 mice [18]. Indeed, while in susceptible BALB/c mice the persistent pattern of infiltration contains PMNs and mononuclear phagocytes, in the resistant C57BL/6 mice, this cellular infiltrate contain besides PMNs, mononuclear phagocytes that rapidly became the dominant population of cells. However, a more recent histopathological study that had looked to the patterns of tissue responses at the site of the infection and in the draining lymph nodes shows no correlation with resistance or susceptibility [19]. The outcome of the lesions and infection depend also on the parasite replication capacity. The transcription of the Macrophage chemoattractant protein-1 (MCP-1/Ccl2) and MIP-1α/Ccl3 that can trigger iNOS activity and promote parasite killing by the host macrophage, is repressed (compared to the one induced by Kp) in C57BL/6 derived macrophages [20]. On the other hand, besides its role as Th1-attracting chemokine, Cxcl9 has also been described as a defensin-like protein with antibacterial activity [21]. An important enhancement of the mRNA coding for this chemokine having a potential anti-*Leishmania* activity could also contribute to the resistant phenotype of C57BL/6 mice.

Thus, strains specific pathogenesis could be due to a difference in the magnitude of the same pathways. However, differentially expressed genes and their corresponding enriched pathways in the macrophages derived from susceptible and resistant mice are potentially the key to understand the different pathologies associated with the two mouse strains.

Among the genes unique to BALB/c derived macrophages we found a set of genes implicated in the p53 signaling pathway. Our data and particularly the mRNA expression of mouse double minute 2 (Mdm2) suggest the activation of this pathway. Indeed, Mdm2 which directly binds to and forms a complex with p53, causes its ubiquitinization and proteasomal degradation, and exports it out of the nucleus, is also a target gene of p53 transcription factor [22].

Our results also show that the induced genes unique to susceptible BMdMs fall into different interconnected pathways. These linked pathways correspond among other to immune response and metabolic pathways (glycolysis and gluconeogenesis). The GENMAPP-CS analysis shows that the p53 pathway is not linked to the others. However, this analysis does not allow the visualization of indirect connections and only considers genes unique to BALB/c derived macrophages. Shared genes may also be interconnected to those selected by GENMAPP-CS and may contribute to the physiopathology of the disease. This could be the case for Glutaminase 2 (Gls2), a p53 target gene [23]. Gls2 catalyzes the hydrolysis of glutamine which feeds the TCA cycle by providing α-ketoglutarate from glutamate and represent an alternative to glucose as the fuel for bioenergetic pathways. Besides cellular energy metabolism, Gls2 regulates antioxidant defense function in cells by increasing reduced glutathione (GSH) levels and decreasing ROS levels, which in turn protects cells from oxidative stress (e.g., H_2_O_2_)-induced apoptosis [23,24]. This gene is heavily transcribed in the macrophages derived from the susceptible mice (Additional file 1: Table S1). Thus, if expressed, this protein may protect the cells from the apoptosis induced by the ROS which may allow the parasite to survive and thus may contribute to the susceptible phenotype. By contrast, in macrophages derived from resistant mice, the transcription of Gls2 is less important. Compared to the transcription induced by killed parasites the one induced by live promastigotes seems to be actively repressed. The relative repression or attenuated transcription of this gene may be in favor of *Leishmania*-infected macrophage elimination by apoptosis and could thus contribute to the resistant phenotype of C57BL/6 mice.

STRING analysis performed to highlight possible direct relationship between the p53 pathway unique to BALB/c and mTOR pathway unique to resistant BMdMs, reveals the Igf1 protein as a major node in this network (Figure 4). The expression of Igf1 another p53 target gene unique to BALB/c derived macrophages, seems to play a role in the susceptible phenotype. Indeed, the protein encoded by this gene has been described as a growth-promoting factor for *Leishmania* promastigotes and amastigotes [25,26] able to induce the activation of arginase and the reciprocal inhibition of NOS2 pathway in BALB/c derived macrophages [27]. Igf1 protein is also able to repress the expression of Vegfa an mTOR target gene. This cytokine which function is still unclear, has been recently detected in the macrophages derived from susceptible lesions but not in the ones derived from resistant lesions [28].

Different arguments assign p53 as a signaling pathway able to direct the macrophage toward a susceptible phenotype. We thus may assume that a signal could allow the preferential activation of one or the other pathway within the macrophage and thus direct the outcome of *Leishmania* disease.

Our results also show that the mTOR signaling pathway unique to resistant mice derived macrophages directly regulates a range of induced pathways unique to resistant BMdMs. These include chemokines, TLR and insulin signaling pathways. However, mTOR could also regulates glycolysis and lipids synthesis through the activation of a transcriptional program affecting metabolic gene targets of sterol regulatory element-binding protein (SREBP1) and hypoxia-inducible factor (HIF1α) known to be activated in *Leishmania* infected macrophages (data not shown) [29]. It has been recently reported [30] that *L. major* subverts the translation machinery of the macrophages through activation of the translational repressor 4EBP1, a mechanism that involves mTOR cleavage and the consequent inhibition of mTORC1. However, in our hands, *L. major* parasite induces the activation of mTOR phosphorylation in macrophages. This phosphorylation is observed 15 min post-infection and is still present 3 hours post-infection (data not shown).

Recent studies highlighted a new metabolic role for p53 transcription factor which is linked to energy metabolism through the regulation of glycolysis and oxidative phosphorylation [31]. The effects of p53 on glycolytic pathways are likely to be cell and context dependent. P53 can be a negative regulator of glycolysis through the activation of TIGAR [32] and the down-regulation of GLUT1 and GLUT4 glucose transporter genes transcription [33], but can also enhance some steps in this pathway such as HK2 [34]. Therefore, p53 and mTOR signaling machineries can clearly cross-talk and coordinately regulate different functions.

Subversion of macrophage signaling pathways is a key strategy used by the parasite to evade microbicidal effector function of these cells [35,36]. *Leishmania* parasite has the capacity to interfere and manipulate the intracellular macrophage signaling pathways. Activation of CD40 pathway has been reported to protect the cell from *Leishmania* infection [37]. A strong CD40 stimulation results in p38MAPK-dependent IL-12 production, whereas a weaker stimulation induces ERK1/2 mediated IL-10 production. During *Leishmania* infection, the level of CD40-induced ERK1/2 phosphorylation and IL-10 production increase, whereas p38MAPK activation and IL-12 production decrease, showing that a single membrane receptor can regulate two counteracting effector functions by modulating two reciprocally signaling pathways [38]. Such reciprocity has been suggested to arise from a signaling threshold that allows preferential activation of one or the other signaling module associated with the receptor [38].

Clearly, a signal is able to regulate two counteracting effector functions by modulating two reciprocally signaling pathways.

## Conclusion

In summary, we identified a global gene expression pattern that was shared and distinct between the macrophages derived from susceptible and resistant mice and showed that susceptibility or resistance to *L. major* may reflect differences in macrophage responses to this parasite.

## Abbreviations

BMdM: Bone marrow derived macrophage; L: *Leishmania*; qRT-PCR: Quantitative real time PCR; GEO: Gene expression omnibus; MIAME: Minimum information about a microarray experiments; KEGG: Kyoto Encyclopedia of genes and genomes.

## Competing interests

The authors declare that they have no competing interests.

## Authors’ contribution

IR carried out the hybridization of mouse microarrays and helped to draft the manuscript. SR and RBO performed BMdM preparation and infection experiments, RNA samples preparation and helped to draft the manuscript. MRA carried out the bioinformatics analysis and drafted the manuscript. DP and BT performed qRT-PCR validation. BR participated and supervised the hybridization of mouse microarrays. LGT performed the design and the coordination of the study, analyzed the result and drafted the manuscript. All authors read and approved the final manuscript.

## Supplementary Material

Additional file 1: Table S1 Gene expression in *Leishmania* infected cells using qRT-PCR. Selected genes up- or down-regulated more than two-fold in *Leishmania* infected BMdM were controlled by qRT-PCR. Changes in mRNA levels are calculated using the 2^-ΔΔ*CT*^ method. The numbers presented for each time point are the average of the three biological replicates. Click here for file

Additional file 2: Table S2 Up and down regulated unique pathways induced by *Leishmania* in resistant and susceptible BMdM. The KEGG pathways enriched with at least four genes and with a p value <0.05 are reported here. Click here for file
