# Supplementary material for: Comparative analysis of resistant and susceptible macrophage gene expression response to Leishmania major parasite
Source: BMC Genomics. 2013 Oct 22;14:723. doi: 10.1186/1471-2164-14-723 (PMC4007596; doi:10.1186/1471-2164-14-723)
Supplement: Additional file 1: Table S1 — Gene expression in Leishmania infected cells using qRT-PCR. Selected genes up- or down-regulated more than two-fold in Leishmania infected BMdM were controlled by qRT-PCR. Changes in mRNA levels are calculated using the 2-ΔΔCT method. The numbers presented for each time point are the average of the three biological replicates. [file 1471-2164-14-723-S1.pdf]

Gene expression in BALB/c *Leishmania* infected macrophages using qRT-PCR

The numbers presented are annotated as 2 -DD Ct=2power-DDCt

2 -DD Ct=2power-DDCt
DDCt=DCt -DCt NI (t: point of the infection)
DeltaCt=Ct gene of interest-Ct of the reference gene.
Please note that 4 reference genes.were used for the qPCR normalization.

KP: BALB/c macrophage infected with killed parasites
NI=Non infected BALB/c macrophages
P: BALB/c macrophages infected with lived parasite

MGI Symbol		P 1hBALB/c-2-DDCt	P 3hBALB/c-2-DDCt	P 6hBALB/c-2-DDCt	P 12hBALB/c-2-DDCt	P 24hBALB/c-2-DDCt	KP 1hBALB/c-2-DDCt	KP 3hBALB/c-2-DDCt	KP 6hBALB/c-2-DDCt	KP 12hBALB/c-2-DDCt	KP 24hBALB/c-2-DDCt
Tfrc	Iron metabolisme	0.891761868	0.987871297	0.326702353	0.304108209	0.636295651	0.985180231	1.451914669	0.570570508	1.332912891	0.8253066
Slc11a2	et pompe vacular proton	0.997526219	2.712666454	1.534268345	1.41157994	1.227298914	1.188811519	3.304836158	1.992740936	1.879739727	0.917330386
Slc40a1		0.57519711	1.221265326	1.4054942	1.116624539	0.446664964	0.633903439	3.817038531	3.529712878	5.382922235	0.424847408
Trf		0.834055331	0.823935514	0.494968688	0.636742	0.398137868	0.746409886	0.86217467	0.511179127	0.821182384	0.4234563
Arg1	Arginine Pathway	0.755111756	1.310101268	5.916779303	1.535726883	0.863264261	0.668989088	1.487773597	0.769929765	0.398154196	3.445385508
Nos2		1.58014697	6.058350306	47.51766645	7.213036212	6.18884776	2.985128253	2.188736581	7.443754198	1.183476315	5.801276637
Txnrd1		2.381744653	4.632100859	2.788801821	2.317238703	0.909449751	1.910900841	8.092207609	2.133433413	4.067237301	0.605415318
Txn1		1.021910135	1.360050292	2.499227962	1.31574584	1.271893905	1.254693516	1.11009803	2.219106899	1.605244345	1.038595107
Glul	Polyamine metabolism	1.001033323	0.532210914	0.437995869	0.597700298	0.523771694	1.011524783	0.613579456	0.577035707	0.906493083	0.622115777
Gls		1.006684542	1.351077095	0.742377985	0.807923631	0.835088884	0.91750632	1.44394963	0.689692014	1.719089412	0.801605882
Azin1		1.264828765	2.321386375	1.37162593	1.030351554	1.288093661	1.178513199	2.763714011	1.468573443	1.823633521	1.111889755
Srm		0.932163815	0.786832434	0.654722478	0.400614071	0.946027592	1.071037938	0.961214289	1.128290645	1.357446965	0.699671072
Slc2a1	Glycolysis, pathway	1.045465171	6.237206396	5.424859726	2.331280338	1.946427129	1.23981082	2.616866663	1.476478996	0.89908614	1.900112712
Gpi1		0.952787682	1.219866553	1.765215312	1.50440621	1.784026032	0.892355205	1.147139161	0.982782563	0.768152459	1.941919415
Pfkl		0.864612391	2.030091846	2.758770446	0.971923723	1.041088346	0.863440049	1.485830968	0.890749753	0.531378772	1.458858964
Pfkp		1.146454223	2.81047049	5.038486451	1.628966197	1.675851328	0.941392273	1.380610792	1.618931657	1.30472356	1.756171513
Aldoa		0.895661449	1.272868457	2.44300263	1.085678292	2.141659561	1.002615134	1.199214722	1.785605963	0.831874328	2.271641883
Aldoc		2.175042739	2.503841664	0.82384438	3.875041246	2.093953154	4.673266301	1.791581077	1.954708868	0.576014686	2.207934055
Tpi1		0.836861046	1.629321907	3.377133396	1.385044664	2.055094877	0.944379306	1.234515899	1.567650732	0.774885248	2.551920408
Eno2		1.26746896	3.324122003	3.158560231	1.337514934	1.941880156	1.025592713	2.784841078	1.816255395	0.983479919	1.788804121
Pkm2		0.909676454	1.295722403	2.322572691	1.156637407	1.797625506	0.927785315	1.101455849	1.722384311	0.933939391	1.838448483
Gapdh		0.964361136	1.243204244	2.367556328	1.267017689	1.975604509	0.978709709	1.083448065	1.567373217	0.824085444	1.962991261
Pfkfb3		1.279124478	2.425770951	2.056269224	1.682231008	1.284955443	1.223570023	1.605955685	1.026341491	0.57366865	0.843682257
Pdk1		0.683282342	1.566469486	2.606966571	1.265509496	1.717975148	0.670830993	1.384760681	1.073085788	0.558091727	3.646469726
Gls2		3.77865E+97	5.2696E-196	3.11548753	4.3773E+195	3.305E-196	1.040396023	0.867140693	3.19525E-98	2.3211E+195	4.0285E-196
Gbe1	strach degradation	0.861982459	1.864094501	8.64700436	2.153517429	2.056734874	0.905709053	1.43013202	4.213766632	1.582564353	1.630363603
Pgm2		0.851560931	2.649303881	4.796966951	1.881657649	1.837375716	0.984785665	1.313421901	1.693536601	0.649670266	2.085475938
Hk1		1.633415323	0.171992195	0.696562237	1.269212968	0.298065575	1.29759635	0.676703341	1.23215393	0.211222132	0.085637807
Hk2		1.490569609	2.403491442	2.647291538	1.084839539	1.517318725	1.455992493	0.538982997	0.698291064	0.252993548	1.566285364
Pgd	pentose phosphate Pathway	0.910371364	0.971085345	1.711174731	0.862349739	0.861670083	1.112126771	1.074985103	1.471326148	1.785315646	0.693526901
Pdha1	Pyruvate vers acetyl-COA	0.944281395	0.953252292	0.911257328	0.693852062	1.23596895	1.006854059	1.057923274	0.88517754	0.785636666	1.226926436
Ldha	Pyruvate vers acetyl-COA	0.87189446	2.116477278	3.983855768	1.223898004	1.963726712	1.022671582	1.332214756	1.754636376	0.691690797	2.410101239
Pcx	Pyruvate vers Oxaloacetate+	0.786519806	0.995182715	1.938312028	0.951481493	0.993475756	0.93319361	1.01556817	2.296448081	1.10484801	0.728087292
Csl	enzym clés du TCA cycle	0.921782798	0.662938372	1.04831243	0.65477675	0.863907747	0.552322543	1.05694966	0.99841424	0.523094135	1.389619994
Idh1	TCA Cycle	0.907770808	0.519604801	0.530153056	0.649694096	0.760876917	0.983461172	0.795833619	0.90418977	0.927651347	0.830513198
Sdhb		0.863756809	0.83719175	0.77208794	0.561907671	0.933645969	0.96062344	0.788206198	0.831631946	0.870411285	1.070303845
Fh1		0.746804918	0.655810088	0.440700111	0.752303044	0.901546202	0.715290738	0.965995752	0.632495666	0.732788068	1.025501783
Tgfbi	Immune Response	1.021392002	0.544831366	0.724494931	1.10604813	0.739279425	1.143559686	0.704596274	0.321751958	0.495988273	0.805327963
Nr4a1		8.694782868	1.981674483	1.507901823	1.684827434	0.382189046	1.366772697	1.645119157	0.245302272	0.187291792	0.568549027
Il1b		2.002321078	0.970344091	0.802100083	0.766114802	0.170352891	1.322511255	1.083422094	0.179210967	0.493475803	0.170845403
Il1rn		2.256296503	3.609394077	6.661817506	4.102284362	2.482516546	1.480173884	4.913577292	3.525437978	3.839254638	1.32632408
Il6ra		0.84007983	0.435898759	0.468255388	0.731543639	0.69668416	0.790489571	0.577852057	0.575582313	0.580658936	0.708717052
Il7r		0.897995684	1.651928984	1.696165663	1.032057487	1.238069669	0.883001575	1.833800463	2.011384296	1.3989223	1.021201161
Tnf		3.492362543	3.971521096	3.179373481	5.45256791	1.271573627	2.024314341	7.057694992	1.81846459	5.480742103	0.867909588
Icam1		1.173991259	1.748244137	1.467115548	1.246741615	0.949347605	1.123628404	1.426316103	0.945008471	1.306724117	0.735082629
Cd40		1.538844104	2.94777212	5.728108222	3.183705008	0.332943858	1.281874108	3.302953517	1.815491803	2.802208233	0.320935158
Cd83		1.813877571	3.815714774	0.912921099	1.074806288	0.498283083	0.977219518	5.012910002	0.78242195	1.079369755	0.576621053
Cd86		1.280158179	1.67136288	1.240957537	1.433519636	0.357523172	0.955628145	2.325417891	0.601233409	0.95687287	0.319584738
Hmgcr	Chholesterol pathway	1.357089074	1.555692223	1.4248981	0.832287188	0.867140396	1.259738759	2.225907025	1.34105222	1.304835104	0.710381254
Sqle		1.329481544	2.522871396	3.117941262	1.453057372	1.4464193	1.37420406	1.869207417	1.452292534	1.247097418	1.350587475
Cyp27a1		0.846888141	0.660293035	0.171129553	0.446472792	0.462208811	0.789867007	0.845118421	0.318366219	0.867173705	0.74717599
Abca1		0.851528713	0.877210509	0.673473811	0.834468791	0.757446467	0.974690609	1.161055283	0.810096352	1.003053924	0.76790447
Scd2		0.939264464	1.008035653	1.779681616	1.867332924	2.871517459	0.864010051	1.035138456	1.228197439	1.137498453	2.767103614
Cd36		0.84873167	1.435616092	2.286641847	1.96686781	2.485393297	0.845080834	2.401570787	4.476978583	5.99473439	3.182110093
Cav1		0.967616371	1.01872289	2.162055788	1.338900112	0.674514253	1.310720052	0.618459059	0.567829123	0.571662608	0.519513118
Lrp12		0.934865301	1.705248114	1.424504735	0.825000367	1.093620734	1.017623987	2.223470607	1.875792722	1.136302932	0.871131297
Acsl1		1.12963661	2.153497527	3.623177327	1.438837169	1.39693591	1.394917292	2.249771271	2.100404342	1.342667893	0.950430968
Fabp4		1.21260142	2.204021555	1.99529042	1.21761525	1.21945301	1.407654148	3.579467548	3.144950006	1.029783339	0.78044223
Lpl		0.6334578	0.87081309	0.92580617	1.684563719	2.948509481	0.684421151	1.988972506	4.390830217	6.086456315	3.566450005
Ppap2b		1.161150519	4.356873825	7.421517281	2.221543668	2.341151559	1.311085405	2.684088207	5.54353341	3.65462805	1.259611063
Dgat2		0.764091376	2.699664261	1.270035435	0.826937386	0.803812453	0.975529846	2.728765336	1.014474135	1.495603238	0.73544299
Agpat5		0.902617768	2.198086763	1.832792868	0.963083862	1.028357933	1.060633521	1.510896672	1.176958744	0.908009418	1.000024549
Cxcl1	Chemokine	6.370862718	4.978059014	2.493257584	4.566144978	0.638346139	3.365193781	16.14809044	1.541292222	4.183515654	0.455544454
Cxcl10		1.236915261	1.017026074	3.10586653	3.981975009	1.234228916	1.674064367	2.493776346	1.965299472	3.572063969	0.864593163
Cxcl2		6.766385701	5.360286081	2.366323115	3.948405057	0.505413405	4.064185494	14.17285335	1.272815889	3.009215653	0.344483969
Cxcl3		2.733631448	2.271567748	3.870072026	2.336944275	0.893434683	2.862593699	7.098930387	2.220993879	6.193346516	0.475931357
Ccl2		5.497783137	1.758849044	0.952948949	2.382981085	0.724222548	3.45325052	5.664821184	0.933702057	1.976136929	0.581629225
Ccl3		4.173247305	2.973677789	1.556676055	2.482720176	1.256079258	4.837191263	14.09296567	2.148647398	2.535231517	0.985631601
Ccl4		4.327464796	4.175401698	2.37537521	3.091213572	0.91452161	4.237537837	11.6386622	1.733356915	2.738237341	0.901329855
Ccr2		0.94778304	0.408600158	0.584168748	0.678832088	0.651855532	0.863221628	0.632224274	0.416299025	0.461056086	1.018410183
Ccrl2		2.132965183	4.313542987	2.69304266	1.509058081	0.776141983	1.701907801	4.68308752	1.507351017	0.815604044	0.62303513
Pla2g4a	Prostaglandine synthesis	1.080155299	2.33603194	4.100643761	1.511345321	1.296215718	1.233239425	1.580593814	1.75441139	0.962081377	1.103815271
Ltb4dh		0.917572038	1.457972323	3.711255947	1.7832729	1.565753026	0.910859222	1.445122792	3.557916123	1.936280436	1.264403266
Ptgds2		0.864727265	1.538998331	0.640016979	0.471031898	0.814959818	0.860033394	1.717892785	0.849761812	1.056479056	1.037053568
Ptger4		2.277980531	1.187974059	0.785826151	1.073349561	1.020920717	2.13859938	2.518215356	0.823048156	1.05609345	0.978423131
Ptges		1.402295521	3.984034112	17.93625866	3.635398039	2.750789671	1.985326448	1.893239158	4.04710071	1.533591416	1.448331734
Ptges3		1.045249304	1.358600854	1.37670592	0.718894348	0.858885369	1.089376701	1.342065421	1.215701258	1.129246806	0.780072661
Ptgs1		0.976846478	0.364524663	0.120984887	0.309950557	0.450079211	0.923427915	0.472399169	0.175542275	0.514190847	0.719830307
Ptgs2		8.396377802	5.349911403	10.16768087	4.934401127	1.894038732	3.217528476	4.732699537	2.033004981	0.953279273	0.800553884
Itgam	Type I phagocytosis mediatec	0.92591313	0.656638692	0.620117604	0.830881707	0.63545381	0.880239438	0.89169568	0.512418837	0.703510649	0.690744344
Mrc1		0.744228047	0.922597529	0.446285601	0.785359895	0.596793337	0.708389631	1.411483323	0.62225558	1.189308439	0.674375175
Fcgr1		0.794382319	0.543530664	1.251024824	1.128553647	0.993449933	0.631361379	0.963728272	1.049441744	0.499626706	1.877498927
Fcgrt		0.789960315	0.619068089	0.340689743	0.372014734	0.520722205	0.697841475	0.759568334	0.378435784	0.9078395	0.891866955
Atp6v0a1	ATPase, H+ transporting, lyso	0.886483336	0.93120239	0.460905775	0.420221437	0.956879503	0.771735254	1.093664322	0.950196029	1.962991828	1.022920305
Atp6v0d1		0.843520723	1.245643875	0.962984011	0.711970811	1.023480891	0.950592934	1.32664129	1.241767791	1.282725526	0.969901683

Gene expression in C57BL/6 Leishmania infected macrophage using qRT-PCR

The numbers presented are annotated as 2 -DD Ct=2power-DDCt

2 -DD Ct=2power-DDCt
DDCt=DCt -DCt NI (for each time point of the infection)
DeltaCt=Ct gene of interest-Ct of the reference gene.
Please note that for the qPCR normalization we used 4 reference genes.

KP: C57BL/6 macrophage infected with killed parasite
NI=Non infected C57BL/6 macrophages
P: C57BL/6 macrophages infected with lived parasite

MGI Symbol		P 1hC57BL/6-2-DDCt	P 3hC57BL/6-2-DDCt	P 6hC57BL/6-2-DDCt	P 12hC57BL/6-2-DDCt	P 24hC57BL/6-2-DDCt	KP 1hC57BL/6-2-DDCt	KP 3hC57BL/6-2-DDCt	KP 6hC57BL/6-2-DDCt	KP 12hC57BL/6-2-DDCt	KP 24hC57BL/6-2-DDCt
Tfrc	Iron metabolisme	1.193837657	1.806641704	0.75530544	0.452739708	0.438455804	1.027130268	1.516862169	1.165712551	1.012827688	0.91666458
Slc11a2	et pompe vacular proton	1.45354132	4.155288799	3.137849699	1.812272625	1.570500697	1.599373715	4.476007644	3.949468001	2.426471993	1.647134463
Slc40a1		0.832574003	1.155965828	1.307719314	1.066785411	0.506896433	1.095008511	4.374578954	5.059997481	3.939495922	1.101962401
Trf		0.735937634	0.688806383	0.50993358	0.263291588	0.256906966	0.786689964	0.80155171	0.566061222	0.323444527	0.359000643
Arg1	Arginine Pathway	1.196329666	1.106846697	5.664003211	19.59405038	9.933932887	1.467763134	0.947731306	0.370522678	0.16164804	2.692956327
Nos2		1.643640745	18.83423351	72.25791644	167.8021143	71.53041963	1.649454711	2.450554671	4.450612051	2.803719621	21.44608646
Txnrd1		5.617338206	11.49178034	4.938719677	1.762911946	0.966908761	3.323759126	9.690738861	6.279967458	3.748596175	1.426018304
Txn1		0.959515988	1.25375207	2.179423025	2.040498524	1.381732094	1.085029111	1.463286738	1.856177915	1.709673743	1.593633633
Glul	Polyamine metabolism	0.723051552	0.452810845	0.396335175	0.423763137	0.477742871	0.89671851	0.638189082	0.623518095	0.703256007	0.679714906
Gls		1.166303895	1.467805102	1.670308495	0.972206712	1.056645361	1.005409865	1.299970754	1.322712943	1.437824616	1.294090339
Azin1		1.787167679	3.628186835	2.118146071	1.59152382	1.175515314	1.487406691	2.546042791	2.702351642	3.07019527	1.414616342
Srm		0.761877876	0.827653466	1.05344896	0.878259237	0.997913496	1.062747722	1.126499495	2.265260683	1.969934849	1.340064554
Slc2a1	Glycolysis, pathway	1.743102109	14.3346067	10.00907155	7.618304749	3.018572077	1.236847031	2.070306211	2.154515285	2.523772513	2.246205621
Gpi1		0.996046787	1.529998689	1.954558705	2.966770509	2.198738832	1.062002442	1.143071647	0.837334902	1.068792564	1.733572693
Pfkl		1.056611487	3.876757292	4.696443815	2.496298429	1.624878299	1.145453717	1.263532991	1.097322113	0.948498294	1.831701183
Pfkp		1.558631631	4.942635155	8.176190157	3.389452102	1.261383238	1.40937367	1.689949341	1.500580189	1.383407564	1.655766422
Aldoa		0.976556215	1.559046275	2.365014043	3.236180514	2.67387434	0.928893971	1.236452371	1.484285635	2.035331445	2.401597144
Aldoc		0.55473667	1.111991235	1.247857981	2.978805136	8.077627774	0.363424898	0.417838789	1.982121524	1.281723068	4.75099877
Tpi1		0.976680603	1.868467431	3.155363578	4.186351161	3.277690388	1.084058103	1.112185551	1.061976371	1.571336184	2.544631357
Eno2		1.682051254	5.880791806	5.231248909	3.574555247	2.715842847	1.249000597	4.294950984	6.704944622	3.900199487	2.629142794
Pkm2		0.963729385	1.503326891	2.00960053	2.814987957	1.801380979	0.932911738	1.182318175	1.40870587	1.733785493	1.728873725
Gapdh		1.034534435	1.581164077	2.438620761	4.025571881	2.508204536	1.003888588	1.13761837	1.225835522	1.642361921	1.904388075
Pfkfb3		2.036429801	6.521959203	3.852151873	2.681536703	1.230956332	1.495751403	2.399353676	1.291934863	1.094072651	0.965616121
Pdk1		1.024433735	1.927214407	3.164732916	3.020872756	2.370319715	0.956845235	0.988631651	0.716358541	1.598345818	2.162051417
Gls2		3.94774E+97	1.68419E-98	7.90686E+97	1.85693E-98	0.736578751	2.72634E+98	8.1234E+97	3.30502E+97	1.6813E-147	0.727073729
Gbe1	strach degradation	0.916325242	1.31640683	5.293794102	5.361303286	2.219408144	1.059485737	1.462777154	4.50170156	4.773727616	2.612022818
Pgm2		0.92939068	2.537270059	4.763602119	4.943256152	2.350687755	1.025736678	1.136400076	0.956149644	1.496280901	2.018851787
Hk1		1.354546966	1.160166674	21.31299225	14.2297328	1.203276267	0.142845459	0.645510514	0.398466405	2.613817732	0.757808381
Hk2		1.515749836	2.97863996	2.96443141	2.610845494	1.25062275	0.940098719	0.212574679	0.469937375	0.645480127	0.894635865
Pgd	pentose phosphate Pathway	0.837871941	1.38181111	1.878314638	1.516999297	0.954953021	0.914390614	1.369776011	2.224527217	2.279643009	1.273157438
Pdha1	Pyruvate vers acetyl-COA	0.931859916	0.898028882	0.81946851	0.929151282	1.010760833	0.919974535	0.935586749	0.880538964	0.989909071	1.186437278
Ldha	Pyruvate vers acetyl-COA	1.03613477	2.228846224	4.052818076	4.054335327	2.702322765	0.949852783	1.167948505	1.222129431	1.567998939	2.20306058
Pcx	Pyruvate vers Oxaloacetate+	0.899922135	1.240599186	3.578891773	1.880498297	1.696580145	1.009434348	1.350670561	3.215103096	4.016104684	2.262229036
Csl	enzym clés du TCA cycle	0.908355121	0.846050343	0.673192224	0.852125059	0.563340201	0.92837259	0.881029155	0.77879674	1.064468248	1.233056722
Idh1	TCA Cycle	0.822238454	0.598682118	0.414614147	0.658343911	0.62472056	1.017410637	1.10080391	0.960863454	0.967465894	0.789179289
Sdhb		0.791385937	0.742698593	0.680294855	0.824934846	0.8242992	0.897226519	0.827748846	0.729685699	1.043442005	1.078782607
Fh1		0.813203737	0.64708348	0.621851174	0.660587542	0.8844615	0.94324672	0.842276508	0.637815767	0.874740932	1.158911179
Tgfbi	Immune Response	0.806033711	0.787771293	0.784214153	1.228398225	1.038261307	0.94349302	0.701637373	0.264396503	0.13268819	0.830380805
Nr4a1		9.768071429	1.244164359	0.82410377	0.148560931	0.206933088	0.734597258	0.155366155	0.100615536	0.051097749	0.238016748
Il1b		3.172397937	2.71236641	1.559710114	0.638512455	0.680850325	1.336171708	0.493977591	0.24437693	0.166255738	0.5552079
Il1rn		2.921971453	10.73469094	10.43049883	8.650109126	4.273568508	1.720763406	5.651768997	8.610517813	8.286082637	3.332900697
Il6ra		0.802208273	0.314392509	0.40830088	0.52433836	0.606598814	0.929981389	0.458384712	0.425442677	0.551459513	0.726531204
Il7r		1.072438561	2.284014933	1.966620723	1.974645602	1.500312815	1.363809841	2.806090749	2.970114914	2.607885297	1.460032854
Tnf		6.363213009	24.65064942	9.922254188	3.497627542	1.721704936	2.653645063	3.938466943	4.411818173	3.251820692	1.848623806
Icam1		1.334038605	4.224475067	3.353446494	1.633420984	1.385009863	1.065273406	1.701099466	1.252438706	1.280720838	1.209084437
Cd40		3.080127347	7.644199426	18.36483145	2.27233218	1.954450889	1.833163967	2.026691733	5.442245671	0.954342868	2.692066721
Cd83		3.978974537	9.419553985	2.740176361	0.633991506	0.722815826	2.043474395	4.578330674	1.841896612	0.622826794	0.79521167
Cd86		1.706022491	2.335282499	1.79702077	1.233426787	1.090913465	1.350027848	1.599469335	1.218072847	0.528320849	0.829088205
Hmgcr	Chholesterol pathway	1.673979275	2.517989179	1.981153159	1.216228035	1.045476041	1.152659049	2.40175113	2.674953384	1.776767099	1.289971478
Sqle		1.482475573	2.853314454	2.576582434	2.644619958	1.737022771	1.056565714	1.209858506	1.291168317	1.758488926	1.782160783
Cyp27a1		0.768775226	0.563118486	0.269680775	0.211071203	0.572384397	0.822775316	0.657613809	0.467361781	0.740589521	0.928038567
Abca1		0.84229159	0.923916098	0.908834023	0.700708879	0.686643581	1.078124783	1.517835034	1.011897914	0.687638829	0.896828584
Scd2		0.982060459	0.901793333	1.813933102	5.076315251	4.70465169	1.147280969	1.107027325	0.893800651	1.960509376	3.685175517
Cd36		1.043899868	2.075840241	2.433476344	2.91433236	1.539106788	1.470184789	4.624577818	7.539560443	9.255245443	3.655796673
Cav1		0.956295461	2.24483657	3.998051576	3.836822657	1.046226342	0.970991676	0.898215382	0.654488047	0.639692105	0.822227293
Lrp12		1.075970358	2.044449013	1.654163779	1.383472292	1.182196619	1.206692013	3.098492909	2.804898859	1.743608631	1.229987937
Acsl1		1.080066393	3.574043227	6.258558541	3.596625011	2.154027497	1.126967439	2.759034187	3.537177826	1.767138868	1.515519589
Fabp4		2.590457055	4.059490729	2.543153865	3.04442636	2.073565088	3.097353042	5.942465682	4.705817648	2.792983563	1.519081387
Lpl		0.800852431	0.688123181	0.896177918	2.461823076	2.20185836	1.229571673	2.505459123	4.266487289	8.34489415	3.560321442
Ppap2b		1.536133659	16.00044824	13.87899549	6.301047418	2.071952961	1.605601367	8.927445986	10.76802431	12.39005805	2.444709992
Dgat2		1.183334817	3.453786132	3.350768656	1.821556928	1.546551633	1.242046483	1.123475243	0.817853163	0.697276976	1.715898441
Agpat5		1.012511528	2.112032571	2.156677438	1.470680387	1.148982374	1.082536623	1.663878398	1.659490553	1.231119879	1.332755836
Cxcl1	Chemokine	13.93400744	37.50463726	10.03459452	3.1584982	2.629973629	5.997491329	9.364086968	5.33616675	2.381256312	3.269339426
Cxcl10		1.289305143	4.159058805	10.77383211	11.24840339	8.604071916	1.122700994	1.693665731	3.437393463	2.125271088	5.065670831
Cxcl2		16.45447776	40.51503317	8.919165598	3.871024441	1.802246899	8.20673954	11.05825367	5.016966889	2.46455484	1.994613427
Cxcl3		3.104142956	15.70746815	9.113111695	4.158333353	1.869872628	1.939904772	5.691898755	3.421811807	2.397257001	2.423892696
Ccl2		7.626575585	7.687718002	3.201288984	3.093153293	1.441199027	5.569439265	4.152477929	1.047285654	1.102045947	1.416792356
Ccl3		9.023455411	19.5682624	5.062010208	2.257752093	1.777200764	8.232016268	18.77184221	6.37371164	3.918026304	1.865954965
Ccl4		8.404109736	16.6251688	5.96462767	2.182286348	1.397999004	6.295666708	8.135503673	3.640837666	2.669956524	1.419625932
Ccr2		0.78257088	0.433224604	0.299707844	0.690807388	1.12054066	0.907853134	0.545405064	0.265968154	0.273616174	0.871164759
Ccrl2		4.80844361	19.380028	9.621168247	3.503265713	1.999022901	2.965343362	7.601662639	4.127582592	1.694257217	1.452064797
Pla2g4a	Prostaglandine synthesis	1.199149611	2.662940911	3.032134499	1.934430303	1.090609782	1.1765363	1.604447593	1.458360189	1.082418136	1.138289481
Ltb4dh		0.935903757	1.43648084	2.589633469	2.912026195	1.628779434	1.076746587	1.49960669	2.36706611	2.752148929	2.088233926
Ptgds2		0.962993746	1.467257501	0.862852232	0.664592577	0.837074732	1.073982759	1.730809167	1.510391011	1.203662771	1.203395472
Ptger4		2.166465606	2.261644356	1.180362847	1.267427962	1.235611326	2.004111117	2.201067415	1.466850747	1.256242152	1.326876564
Ptges		1.24513025	13.13229432	23.67359799	9.915590903	3.805114852	1.100358906	2.509668247	3.161663329	1.793707418	2.746601296
Ptges3		0.91716614	1.524781038	1.455734324	1.200161662	0.916455456	0.970789785	1.356961699	1.615673201	1.468243499	1.181234856
Ptgs1		0.744957486	0.289912696	0.106970909	0.147103698	0.296383424	0.747317131	0.285088154	0.107415226	0.147576637	0.418838945
Ptgs2		7.810491478	37.10355241	12.15253527	5.164237736	3.524926886	1.602620126	2.234225803	1.694105534	1.450839487	2.599418939
Itgam	Type I phagocytosis mediatec	0.882732569	0.761582421	0.609490406	0.769924428	0.849291471	0.920175426	0.821027054	0.672379239	0.464147553	0.730605321
Mrc1		0.83396115	0.994992174	0.763656764	0.51104834	0.706677028	1.087679682	1.369751484	0.972189865	0.889423672	1.088405888
Fcgr1		0.863165037	0.966370788	0.926772463	1.835483845	1.083261586	0.9648417	0.997470906	0.839775391	0.753833989	1.805233972
Fcgrt		0.757008638	0.602146074	0.463012725	0.319767174	0.474177792	0.842003129	0.691597711	0.560934964	0.415446973	0.783500355
Atp6v0a1	ATPase, H+ transporting, lyso	0.887018849	0.93272827	0.658943687	0.56404692	0.793699609	0.971843445	1.279473263	2.100035549	2.241599828	1.264969412
Atp6v0d1		0.945595451	1.398571247	1.288270381	1.157852985	0.988696753	0.993717378	1.619774897	1.887033999	1.799807882	1.228887203
