# Supplementary material for: Comparative analysis of resistant and susceptible macrophage gene expression response to Leishmania major parasite
Source: BMC Genomics. 2013 Oct 22;14:723. doi: 10.1186/1471-2164-14-723 (PMC4007596; doi:10.1186/1471-2164-14-723)
Supplement: Additional file 2: Table S2 — Up and down regulated unique pathways induced by Leishmania in resistant and susceptible BMdM. The KEGG pathways enriched with at least four genes and with a p value <0.05 are reported here. [file 1471-2164-14-723-S2.pdf]

UPREGULATED - BalbC - Uniques

Category	Term	PValue	LOG10(pvalue)	Genes	Pop Hits	Fold Enrichment	Bonferroni	Benjamini	FDR
KEGG_PATHWAY	mmu04115:p53 signaling pathway	6.10E-07	4.322759305	BID, CDKN1A, GADD45G, SERPINE1, IGF1, GADD45B, CCNG2, GADD45A	69	15.1198946	4.76E-05	4.76E-05	6.45E-04
KEGG_PATHWAY	mmu04010:MAPK signaling pathway	2.55E-06	4.002378455	DUSP4, ATF4, MAPKSP1, DUSP1, PDGFA, MAP3K1, GADD45G, TRAF6, GADD45B, RAPGEF2, GADD45A, IL1A	265	5.905317324	1.99E-04	9.95E-05	0.00269754
KEGG_PATHWAY	mmu05215:Prostate cancer	0.004379594	0.967185116	HSP90AB1, CDKN1A, ATF4, PDGFA, IGF1	90	7.244949495	0.28990648	0.10784869	4.5366934
KEGG_PATHWAY	mmu00040:Pentose and glucuronate interconversions	0.006947002	0.895839529	AKR1B8, UGDH, UGP2	17	23.01336898	0.41943709	0.12710437	7.10872565
KEGG_PATHWAY	mmu05218:Melanoma	0.015731975	0.729828991	CDKN1A, PDGFA, MET, IGF1	71	7.346991037	0.70970245	0.18628205	15.4420117
KEGG_PATHWAY	mmu04722:Neurotrophin signaling pathway	0.01568973	0.660299971	IRAK2, MAGED1, ATF4, MAP3K1, TRAF6	130	5.015734266	0.70872898	0.2186251	15.4036142
KEGG_PATHWAY	mmu05200:Pathways in cancer	0.031828579	0.519099006	HSP90AB1, BID, CDKN1A, PDGFA, MET, IGF1, TRAF6	323	2.826203209	0.91978119	0.30262235	28.9759004
KEGG_PATHWAY	mmu00520:Amino sugar and nucleotide sugar metabolism	0.042549634	0.461502655	CYB5R3, UGDH, UGP2	44	8.891528926	0.96634349	0.34553922	36.8677038

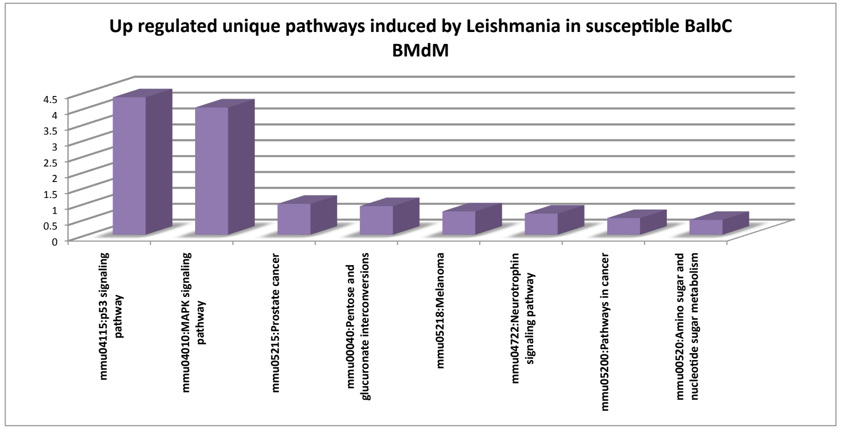

DOWNREGULATED - BalbC - Uniques

Category	Term	PValue	LOG10(pvalue)	Genes	Pop Hits	Fold Enrichment	Bonferroni	Benjamini	FDR
KEGG_PATHWAY	mmu04330:Notch signaling pathway	8.71E-08	5.178933183	NCSTN, NOTCH2, NOTCH1, HDAC2, EP300, CREBBP, LFNG, NCOR2	50	19.95826087	6.62E-06	6.62E-06	9.17E-05
KEGG_PATHWAY	mmu05211:Renal cell carcinoma	9.31E-07	4.451426881	EP300, HIF1A, SOS2, PIK3CD, CREBBP, GAB1, HGF, FLCN	70	14.25590062	7.07E-05	3.54E-05	9.79E-04
KEGG_PATHWAY	mmu04062:Chemokine signaling pathway	4.71E-04	1.92621864	CXCL16, SOS2, PIK3CD, CX3CR1, GNG2, CSK, PRKCB, ELMO1	182	5.4830387	0.03513543	0.01185172	0.49401514
KEGG_PATHWAY	mmu05016:Huntington's disease	0.002804579	1.284304803	HDAC2, EP300, POLR2E, CREBBP, ATP5A1, NDUFA10, POLR2B	183	4.771442148	0.19220542	0.05196312	2.91216177
KEGG_PATHWAY	mmu04142:Lysosome	0.013613547	0.859255157	AP1M1, LAPTM4A, CTSE, CTSC, MANBA	119	5.241139934	0.64715861	0.13827537	13.4319595
KEGG_PATHWAY	mmu04722:Neurotrophin signaling pathway	0.018299606	0.84040935	SOS2, PIK3CD, GAB1, CSK, ARHGDIB	130	4.797658863	0.75430215	0.1444078	17.6630829
KEGG_PATHWAY	mmu04320:Dorso-ventral axis formation	0.012575414	0.82941377	NOTCH2, NOTCH1, SOS2	22	17.00988142	0.61779251	0.14811063	12.4683947
KEGG_PATHWAY	mmu04720:Long-term potentiation	0.017116442	0.820247816	EP300, CREBBP, PPP1R12A, PRKCB	70	7.127950311	0.73074901	0.15126978	16.6128297
KEGG_PATHWAY	mmu05200:Pathways in cancer	0.01198517	0.776086591	HDAC2, EP300, HIF1A, SOS2, PIK3CD, CREBBP, HGF, PRKCB	323	3.089514066	0.60003388	0.1674609	11.9162304
KEGG_PATHWAY	mmu04662:B cell receptor signaling pathway	0.024337913	0.767591605	FCGR2B, SOS2, PIK3CD, PRKCB	80	6.236956522	0.84627071	0.17076875	22.8390035
KEGG_PATHWAY	mmu04012:ErbB signaling pathway	0.030234883	0.718668221	SOS2, PIK3CD, GAB1, PRKCB	87	5.735132434	0.9030253	0.19113128	27.607744
KEGG_PATHWAY	mmu05215:Prostate cancer	0.032973766	0.718235053	EP300, SOS2, PIK3CD, CREBBP	90	5.543961353	0.92178187	0.19132202	29.7305301
KEGG_PATHWAY	mmu04666:Fc gamma R-mediated phagocytosis	0.040891924	0.664382521	PTPRC, FCGR2B, PIK3CD, PRKCB	98	5.091393079	0.95812704	0.21657957	35.5545744
KEGG_PATHWAY	mmu00071:Fatty acid metabolism	0.04811685	0.629187189	ACADM, ACSL3, ACOX3	45	8.315942029	0.97643016	0.23486203	40.4837579

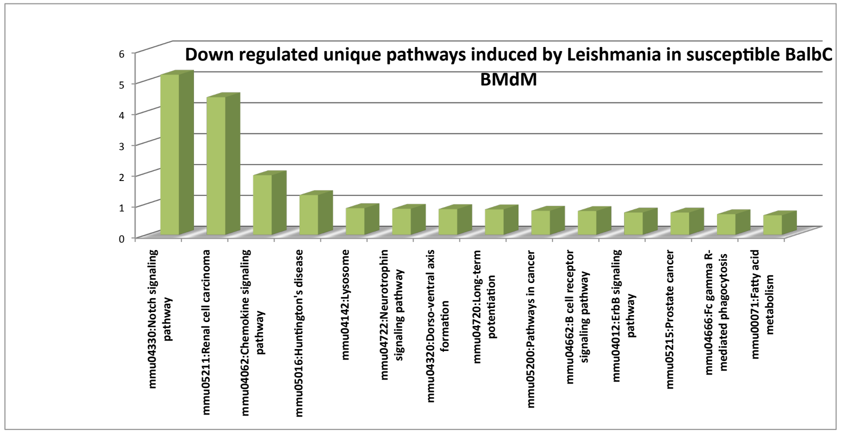

UPREGULATED - C57 - Uniques

Category	Term	PValue	LOG10(pvalue)	Genes	Fold Enrichme	Bonferroni	Benjamini	FDR
KEGG_PATHWAY	mmu00190:Oxidative phosphorylation	0.001594496	1.462208364	ATP6V0E, NDUFC1, ATP5G3, COX5B, COX6C	9.19551282	0.099964214	0.03449782	1.61833904
KEGG_PATHWAY	mmu04150:mTOR signaling pathway	0.001221478	1.403063759	EIF4EBP1, VEGFA, RHEB, PIK3R5	17.7098765	0.077499027	0.03953086	1.24187531
KEGG_PATHWAY	mmu05010:Alzheimer's disease	0.00540468	1.160920923	HSD17B10, NDUFC1, ATP5G3, COX5B, COX6C	6.56822344	0.300700601	0.06903655	5.39023097
KEGG_PATHWAY	mmu04660:T cell receptor signaling pathway	0.001112194	1.149884994	NCK2, NFKBIE, NCK1, PIK3R5, TEC	10.1306497	0.070813328	0.07081333	1.13133507
KEGG_PATHWAY	mmu04012:ErbB signaling pathway	0.004788732	1.118338933	NCK2, EIF4EBP1, NCK1, PIK3R5	10.9923372	0.271534871	0.07614845	4.78945635
KEGG_PATHWAY	mmu04910:Insulin signaling pathway	0.016951417	0.827174437	EIF4EBP1, RHEB, PIK3R5, PRKX	6.92995169	0.676444497	0.1488763	16.0378242
KEGG_PATHWAY	mmu05012:Parkinson's disease	0.015360849	0.805286374	NDUFC1, ATP5G3, COX5B, COX6C	7.19047619	0.640011511	0.15657183	14.6384247
KEGG_PATHWAY	mmu05211:Renal cell carcinoma	0.031469876	0.634750862	VEGFA, PIK3R5, TCEB1	10.2464286	0.878810005	0.23187244	27.8867294
KEGG_PATHWAY	mmu05016:Huntington's disease	0.035358425	0.634476494	NDUFC1, ATP5G3, COX5B, COX6C	5.22586521	0.907070329	0.23201898	30.7927536
KEGG_PATHWAY	mmu04210:Apoptosis	0.046792917	0.566784382	PIK3R5, BIRC3, PRKX	8.24425287	0.957698163	0.27115375	38.7364234
KEGG_PATHWAY	mmu04620:Toll-like receptor signaling pathway	0.058953052	0.514978537	IKBKE, CCL3, PIK3R5	7.24494949	0.981872197	0.30550721	46.2731444

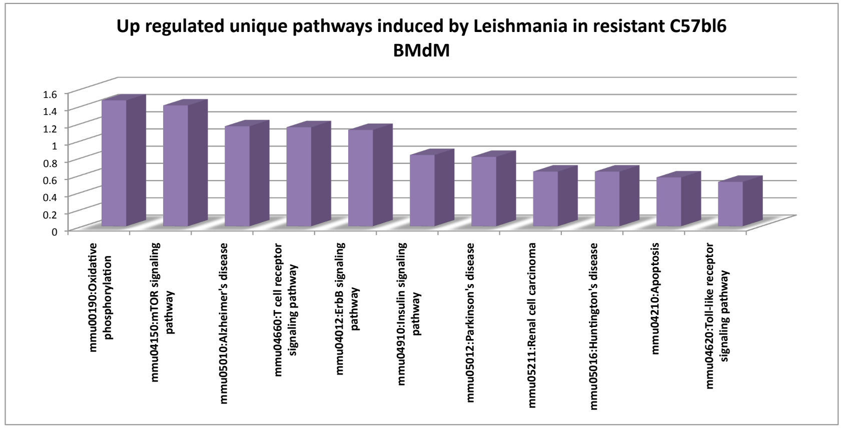

Up regulated unique pathways induced by Leishmania in resistant C57bl6 BMdM

DOWNREGULATED - C57 - Uniques

Category	Term	PValue	LOG10(pvalue)	Genes	Fold Enrichme	Bonferroni	Benjamini	FDR
KEGG_PATHWAY	mmu04144:Endocytosis	1.64E-13	10.79784986	CLTA, USP8, TGFBR1, AP2S1, TGFBR2, ASAP1, EPS15, RAB11FIP5, SMAP2, DAB2, AP2B1, CHMP1A, AP2A2, RAB31, WWP1, VPS24, PDCD6IP, HSPA8, CSF1R, EHD4	8.87685644	1.59E-11	1.59E-11	1.81E-10
KEGG_PATHWAY	mmu04142:Lysosome	4.04E-05	2.708656281	GNS, CLTA, AP1S2, LGMN, ATP6V0A1, PPT2, CTSH, ASAH1, GLB1	6.78072479	0.003907948	0.00195589	0.04453776
KEGG_PATHWAY	mmu04010:MAPK signaling pathway	1.20E-04	2.412759412	MAPK1, RPS6KA3, TGFBR1, TGFBR2, PRKACA, PAK1, STK4, RASA1, HSPA8, PPP5C, AKT2, MAP2K5	4.05990566	0.011552657	0.00386581	0.13211189
KEGG_PATHWAY	mmu05016:Huntington's disease	0.003660614	1.070106593	NDUFA4, SDHB, CLTA, AP2B1, AP2A2, AP2S1, COX4I1, ATP5J	3.91939891	0.299338974	0.08509292	3.96633561
KEGG_PATHWAY	mmu05220:Chronic myeloid leukemia	0.00938462	0.776851269	MAPK1, TGFBR1, TGFBR2, ABL1, AKT2	5.8984375	0.599325734	0.1671663	9.88237503
KEGG_PATHWAY	mmu04914:Progesterone-mediated oocyte maturation	0.013767186	0.757520583	MAPK1, RPS6KA3, PDE3B, PRKACA, AKT2	5.27389706	0.739380758	0.17477504	14.1857752
KEGG_PATHWAY	mmu04722:Neurotrophin signaling pathway	0.013599409	0.702072116	IRAK4, MAPK1, RPS6KA3, ABL1, AKT2, MAP2K5	4.13798077	0.735044835	0.19857651	14.0245324
KEGG_PATHWAY	mmu05200:Pathways in cancer	0.023930528	0.638724156	MAPK1, ITGA6, TGFBR1, TGFBR2, FH1, ABL1, STK4, AKT2, CSF1R	2.49816176	0.904581892	0.22976075	23.4556759
KEGG_PATHWAY	mmu04666:Fc gamma R-mediated phagocytosis	0.022108187	0.624452709	MAPK1, VAV3, ASAP1, PAK1, AKT2	4.57429847	0.885657262	0.2374364	21.8637154
KEGG_PATHWAY	mmu04810:Regulation of actin cytoskeleton	0.030868746	0.581290124	ITGAL, MAPK1, VAV3, ITGA6, PAK1, PIP4K2A, FGD3	2.8921371	0.952234738	0.26224661	29.2505442
KEGG_PATHWAY	mmu00020:Citrate cycle (TCA cycle)	0.044989342	0.536556418	SDHB, ACO1, FH1	8.67641129	0.988497369	0.29069903	39.8303271
KEGG_PATHWAY	mmu05212:Pancreatic cancer	0.044039376	0.515484281	MAPK1, TGFBR1, TGFBR2, AKT2	4.98090278	0.987332807	0.30515165	39.1665168
KEGG_PATHWAY	mmu05211:Renal cell carcinoma	0.041044125	0.510083812	MAPK1, FH1, PAK1, AKT2	5.12321429	0.982842005	0.30896991	37.0296738
KEGG_PATHWAY	mmu00190:Oxidative phosphorylation	0.053750534	0.497506491	NDUFA4, SDHB, COX4I1, ATP6V0A1, ATP5J	3.44831731	0.995295128	0.31804862	45.6491089

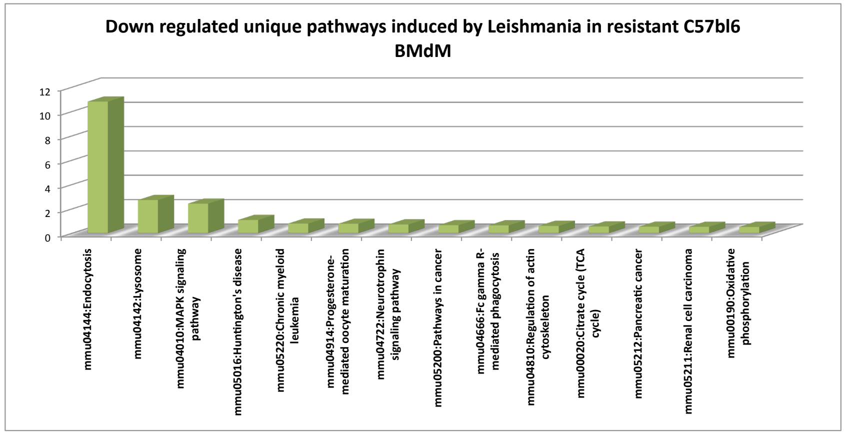

Down regulated unique pathways induced by Leishmania in resistant C57bl6 BMdM
